# The influence of animal species, gender and tissue on the structural, biophysical, biochemical and biological properties of collagen sponges

**DOI:** 10.1007/s10856-020-06485-4

**Published:** 2021-01-21

**Authors:** Anna Sorushanova, Ioannis Skoufos, Athina Tzora, Anne Maria Mullen, Dimitrios I. Zeugolis

**Affiliations:** 1 Regenerative, Modular & Developmental Engineering Laboratory (REMODEL), Biomedical Sciences Building, National University of Ireland Galway (NUI Galway), Galway, Ireland; 2 Science Foundation Ireland (SFI) Centre for Research in Medical Devices (CÚRAM), Biomedical Sciences Building, National University of Ireland Galway (NUI Galway), Galway, Ireland; 3 Laboratory of Animal Science, Nutrition and Biotechnology, School of Agriculture, University of Ioannina, Arta, Greece; 4 Teagasc, Food Research Centre, Ashtown, Dublin Ireland; 5 Regenerative, Modular & Developmental Engineering Laboratory (REMODEL), Faculty of Biomedical Sciences, Università della Svizzera Italiana (USI), Lugano, Switzerland

## Abstract

Although collagen type I is extensively used in biomedicine, no study to-date has assessed how the properties of the produced scaffolds are affected as a function of species, gender and tissue from which the collagen was extracted. Herein, we extracted and characterised collagen from porcine and bovine, male and female and skin and tendon tissues and we subsequently fabricated and assessed the structural, biophysical, biochemical and biological properties of collagen sponges. All collagen preparations were of similar purity and free-amine content (*p* > 0.05). In general, the porcine groups yielded more collagen; had higher (*p* < 0.05) denaturation temperature and resistance to enzymatic degradation; and lower (*p* < 0.05) swelling ratio and compression stress and modulus than the bovine groups of the same gender and tissue. All collagen preparations supported growth of human dermal fibroblasts and exhibited similar biological response to human THP-1 monocytes. These results further illustrate the need for standardisation of collagen preparations for the development of reproducible collagen-based devices.

**Figure:**
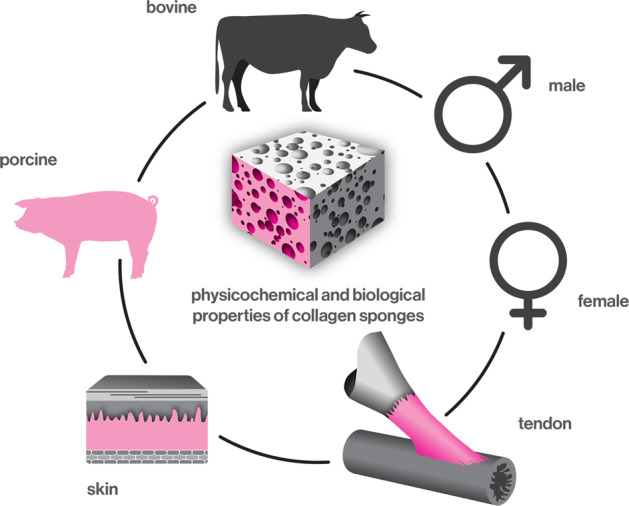
Assessment of the physicochemical and biological properties of collagen sponges as a function of animal species (bovine versus porcine), gender (male versus female) and tissue (skin versus tendon).

Assessment of the physicochemical and biological properties of collagen sponges as a function of animal species (bovine versus porcine), gender (male versus female) and tissue (skin versus tendon).

## Introduction

The term collagen encompasses a large family of proteins with 29 subtypes [1]. Among them, collagen type I is the most abundant in mammalian tissues (e.g. 85–90% in skin [2], 65–80% in tendon [3]). This prevalence in tissues coupled with numerous inherent properties (e.g. cell recognition signals, physiological biodegradability, low antigenicity) makes it the material of choice for biomedical applications [4]. Further, advancements in scaffold fabrication and functionalisation technologies allow the development of three-dimensional implantable devices with clinical indication-specific properties and capacity to deliver a broad range of cells and bioactive molecules in a controlled and localised fashion [5, 6].

Despite the significant advancements in the field, collagen remains an animal by-product and, as such, variability is frequently encountered between different collagen preparations, as a function of species, tissue and extraction method [7, 8], which subsequently influence the properties and performance of the produced scaffolds [9–11]. For example, the properties of extruded collagen fibres have been shown to be species (bovine Achilles tendon versus rat tail tendon) and extraction method (acid versus pepsin) dependent [12]. Yet again, no study to-date has assessed whether the properties of collagen scaffolds depend on the species, gender and tissue from which the collagen is extracted.

In this study, collagen sponges were fabricated and their properties were correlated to the origin of collagen (porcine versus bovine, male versus female and skin versus tendon tissues). Porcine and bovine skin and tendon tissues were selected, as the vast majority of collagen used in biomedicine is extracted from these species and tissues [13]. Pepsin extraction was used, as it results in high yield (cleavage of even mature cross-links) and with reduced immunogenicity and antigenicity (removal of antigenic p-determinant located at the non-helical ends) collagen preparations [14, 15]. Male and female tissues were selected, as recent data have shown gender-dependant disease and injury disposition and differences in, for example, mechanical, structural and compositional properties of tendon [16–21] and skin [22–26].

## Materials and methods

### Materials

Porcine and bovine, male and female and skin and tendon tissues were collected from a local abattoir and transferred to the laboratory on ice. The following abbreviations are used throughout this manuscript: PMS: Porcine male skin, PMT: Porcine male tendon, PFS: Porcine female skin, PFT: Porcine female tendon, BMS: Bovine male skin, BMT: Bovine male tendon, BFS: Bovine female skin, BFT: Bovine female tendon. SilverQuest™ kit was purchased from Thermo Fisher Scientific (UK). Quant-iT™ PicoGreen^®^ dsDNA Reagent was purchased from Invitrogen (Bio Sciences Ltd., Ireland). Human adult dermal fibroblasts and human derived leukaemic monocytes (THP-1) were purchased from ATCC (UK). All chemicals, cell culture media and reagents were purchased from Sigma-Aldrich (Ireland), unless otherwise stated.

### Collagen extraction and yield analysis

Collagen type I was extracted using the acetic acid/pepsin protocol from porcine and bovine, male and female and skin and tendon tissues [12, 14]. Briefly, porcine and bovine tissues (200 g) were cut into small pieces (1 × 1 × 1 cm^3^) using a scalpel and the weight was recorded. Tissue pieces were washed three times for 2 h each in salt solutions (3.7 mM Na_2_HPO_4_, 0.35 mM KH_2_PO_4_, 51 mM NaCl). Tissue pieces were then suspended in 0.5 M acetic acid for 48 h under stirring at 4 °C. During the process, swollen tissue pieces were sieved, blended and re-suspended in the acetic acid solution. 2 g of pepsin (1 g pepsin per 100 g tissue) was added to the acetic acid solution at room temperature for 1 h and then the solution was transferred at 4 °C for 48 h under stirring. The collagen solution was then filtered through a sieve with pore diameter of 250 µm. 0.9 M NaCl was added to the filtered solution, stirred manually every 2 h for 8 h and then left static overnight. The next day, precipitated collagen was collected from the top of the solution and re-suspended in 1 M acetic acid overnight at 4 °C. The solution was then centrifuged at 8000 rpm for 20 min at 4 °C and the supernatant was collected and subjected to a second salt-precipitation and re-suspended in minimum required volume of 1 M acetic acid to produce a concentrated collagen solution. Once fully suspended, the collagen solution was dialysed four times (the first 3 times every 2 h and the last time overnight) against 1 mM acetic acid at 4 °C. The final collagen solutions, at concentration of 5 mg/ml, were stored at 4 °C until use. Yield was calculated as % of original weight (200 gr).

### Collagen purity

Sodium dodecyl sulphate polyacrylamide gel electrophoresis (SDS-PAGE) was performed to assess the purity of the extracted collagen [27]. Briefly, collagen samples were freeze-dried and then dissolved in 0.5 M acetic acid at concentration of 0.1 mg/ml. Collagen samples were neutralised by the addition of 1 N NaOH. Collagen samples (40 µl) were transferred to Eppendorf tubes and 10 µl of the sample buffer (×5) and 34 µl distilled water were added. Bovine (calf hide) collagen type I (Symatese, Biomateriaux, France) was used as a standard at a concentration of 0.1 mg/ml. Samples and standard were heated at 95 °C for 5 min and then loaded onto 3% stacking and 5% separation gels. Gels were run (50 V for ~30 min for the stacking gel and 120 V for ~60 min for the separation gel) using Mini-Protean 3 system (Bio-Rad Laboratories, UK). Gels were stained using SilverQuest™ kit (Thermo Fisher Scientific, UK) following the manufacturer’s instructions.

### Fabrication of collagen sponges

To fabricate collagen sponges, collagen solutions at 5 mg/ml were pipetted into well plates, frozen at −80 °C overnight and freeze-dried (Freezone 4.5 L, Labconco, USA) for 24 h. 24 well plates were used to fabricate sponges for stability analysis with 2 ml of collagen per well and 48 well plates were used to fabricate sponges for biological analysis with 250 µl per well.

### Structural characterisation

The structure of produced collagen type I sponges was visualised using scanning electron microscopy (SEM, Hitachi S-4700, Japan). Adhesive carbon tabs were used on top of SEM specimen stubs. Collagen sponges were cut horizontally and stuck onto carbon tabs. Collagen sponges were gold-coated prior to SEM imaging at 25 mA current for 5 min. Pore diameter was measured using ImageJ software (National Institutes of Health, USA).

### Quantification of free amines

Free-amine groups were determined using the ninhydrin assay [28]. Briefly, 3 mg of freeze-dried samples were added in 1 ml ninhydrin buffer and incubated at 100 °C for 10 min. After the samples were cooled down at room temperature, 50% of isopropanol was added and the absorbance was measured at 570 nm. Free-amine groups were quantified by interpolating values from a linear standard curve of known concentrations of glycine.

### Enzymatic stability analysis

Enzymatic degradation of collagen sponges was assessed using collagenase type I assay [29]. Briefly, 5 mg of freeze-dried samples were added in 1 ml of collagenase solution. The samples were incubated for 3, 6, 9, 12 and 24 h at 37 °C. The supernatants were then collected, freeze-dried overnight and weighed.

### Thermal stability and swelling analyses

Denaturation temperature of the collagen sponges was analysed using differential scanning calorimetry (DSC-60, Shimadzu, Japan) [30]. The collagen sponges were hydrated overnight at room temperature in 0.01 M phosphate-buffered saline (PBS). The sponges were then removed from the PBS and quickly blotted on a filter paper. The sponges were then hermetically sealed in aluminium pans. Heating was carried out at a raising temperature rate of 10 °C/min within temperature range of 20–70 °C. An empty aluminium pan was used as reference. The endothermic transition was recorded as a typical peak and denaturation temperature was defined as the temperature of maximum power absorption during denaturation (peak temperature).

For swelling determination, collagen sponges were incubated in PBS overnight and the next day were quickly blotted using filter paper. Swelling ratio was calculated using the following equation: Swelling (%) = [(Ww − Wd)/(Wd)] × 100, where Ww and Wd refer to the average wet weight and dry weight of the sponges, respectively.

### Mechanical stability analysis

Compression test was carried out using an electromechanical testing machine (Z2.5, Zwick, Germany). The collagen sponges were tested in the dry state, as wet sponges were collapsing. Compression stress and modulus values were calculated as follows: compressive stress was defined as the force at 70% compression divided by the original cross-sectional area and modulus was defined as the slope of the stress-strain (deformation) curve at the elastic deformation region (Young’s modulus).

### Dermal fibroblast culture and analysis

Human adult dermal fibroblasts were used between passages 3 and 5. Collagen sponges were sterilised prior to seeding with UV for 2 h. The cells were seeded onto collagen sponges at a density of 30,000 cells per cm^2^ in 48 well plates. The cells were cultured for 3, 5 and 7 days in Dulbecco’s Modified Eagle Medium, supplemented with 1% penicillin streptomycin and 10% foetal bovine serum at 37 °C and 5% CO_2_. Media were changed every 2 days. Cell proliferation was assessed using PicoGreen^®^ dsDNA assay kit after 3, 5 and 7 days in culture, according to manufacturer’s protocol. Metabolic activity was assessed using the alamarBlue^®^ assay (Thermo Fisher Scientific, UK) after 3, 5 and 7 days in culture, according to manufacturer’s protocol. Cell viability was assessed with calcein AM (Thermo Fisher Scientific, UK) and ethidium homodimer I (Thermo Fisher Scientific, UK) staining after 3, 5 and 7 days in culture, according to the manufacturer’s protocol. The cells were visualised under Andor Revolution Spinning Disk Confocal Microscope (Olympus IX81, Japan). Nuclei were stained with 4′,6-diamidino-2-phenylindole (DAPI), whilst cytoskeleton was stained with rhodamine phalloidin based on established protocols. Briefly, media were removed and sponges were washed three times with Hank’s Balanced Salt Solution prior to staining. Cells were fixed with 2% paraformaldehyde, permeabilised with 0.2% Triton X-100 and then stained with DAPI and rhodamine. The sponges were imaged using Andor Revolution Spinning Disk Confocal Microscope (Olympus IX81, Japan). Cell morphometric analysis was conducted with ImageJ software (National Institutes of Health, USA). The total area and aspect ratio (the ratio of the major axis divided by the minor axis of each nuclei based on a fitted ellipse) of the nuclei were assessed. For cell viability and morphometric analysis, for each experimental group, cells on three sponges were analysed by taking five images per sponge (15 images in total were analysed per experimental group).

### Monocyte culture and analysis

Human derived leukaemic monocyte cells (THP-1) were seeded onto TCP and collagen sponges at a density of 50,000 cells per cm^2^ in 48 well plates. Collagen sponges were sterilised prior to seeding with UV for 2 h. Mature macrophage-like state was induced by treating them with phorbol 12-myristate 13-acetate (PMA) at concentration of 100 ng/ml for 24 h, at 37 °C and 5% CO_2_. The differentiation media was removed and replaced by activation media and the cells were incubated for 48 h, at 37 °C and 5% CO_2_. Activated control was induced with 100 ng/ml of lipopolysaccharide (LPS). Cell metabolic activity, proliferation and viability was assessed at day 1 and day 2, as described above (Section “Dermal fibroblast culture and analysis”).

### Statistical analysis

Statistical analysis was performed using SPSS (version 20.0, IBM SPSS Statistics, IBM Corporation, USA). All values are expressed as mean values ± standard deviation (SD). One-way analysis of variance (ANOVA) for multiple comparisons was employed, after confirming the following assumptions: (a) the distribution from which each of the samples was derived was normal; (b) and the variances of the population of the samples were equal to one another. Nonparametric statistics were used when either one or both of the above assumptions were violated and consequently Kruskal–Wallis test for multiple comparisons was carried out. Statistical significance was accepted at *p* < 0.05.

## Results

### Collagen purity and yield

**Fig. 1:**
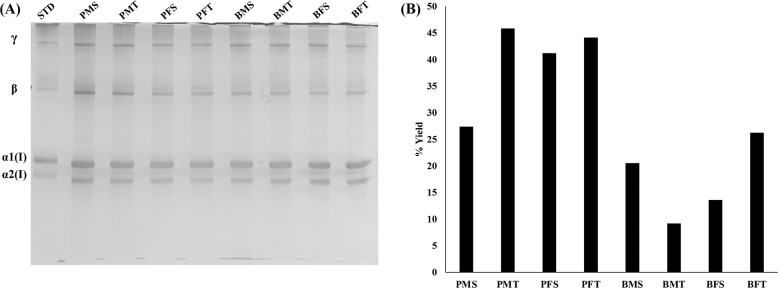
**A** SDS-PAGE revealed that all collagen preparations were of similar purity. STD: 0.1 mg/ml bovine (calf hide) collagen type I (Symatese, Biomateriaux, France). **B** More collagen was extracted from the porcine than the bovine groups of the same gender and tissue

SDS-PAGE revealed that all collagen preparations exhibited typical collagen type I electrophoretic mobility and purity (Fig. 1A). The porcine groups yielded more collagen than the bovine groups of the same gender and tissue (Fig. 1B).

### Structural analysis

**Fig. 2:**
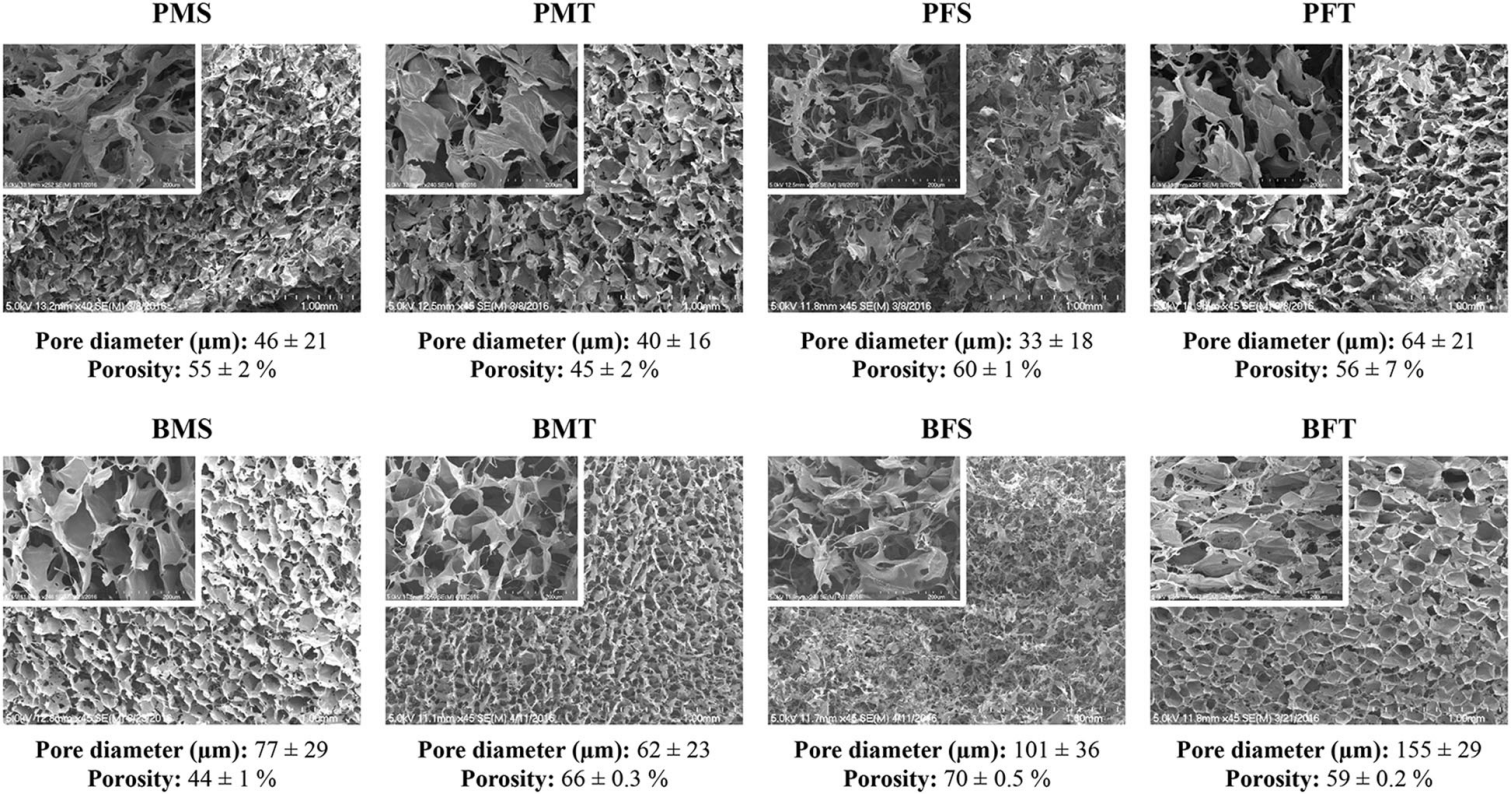
SEM and supplementary porosity analysis revealed that the bovine groups exhibited significantly higher (*p* < 0.005) pore diameter than the porcine groups of the same gender and tissue. *N* = 20

SEM and complementary porosity analysis (Fig. 2) revealed that the bovine groups showed a significantly higher (*p* < 0.005) pore diameter than the porcine groups of the same gender and tissue. No correlation was observed between male and female and skin and tendon tissues across the different species with respect to porosity.

### Free amine and resistance to enzymatic degradation analyses

**Fig. 3:**
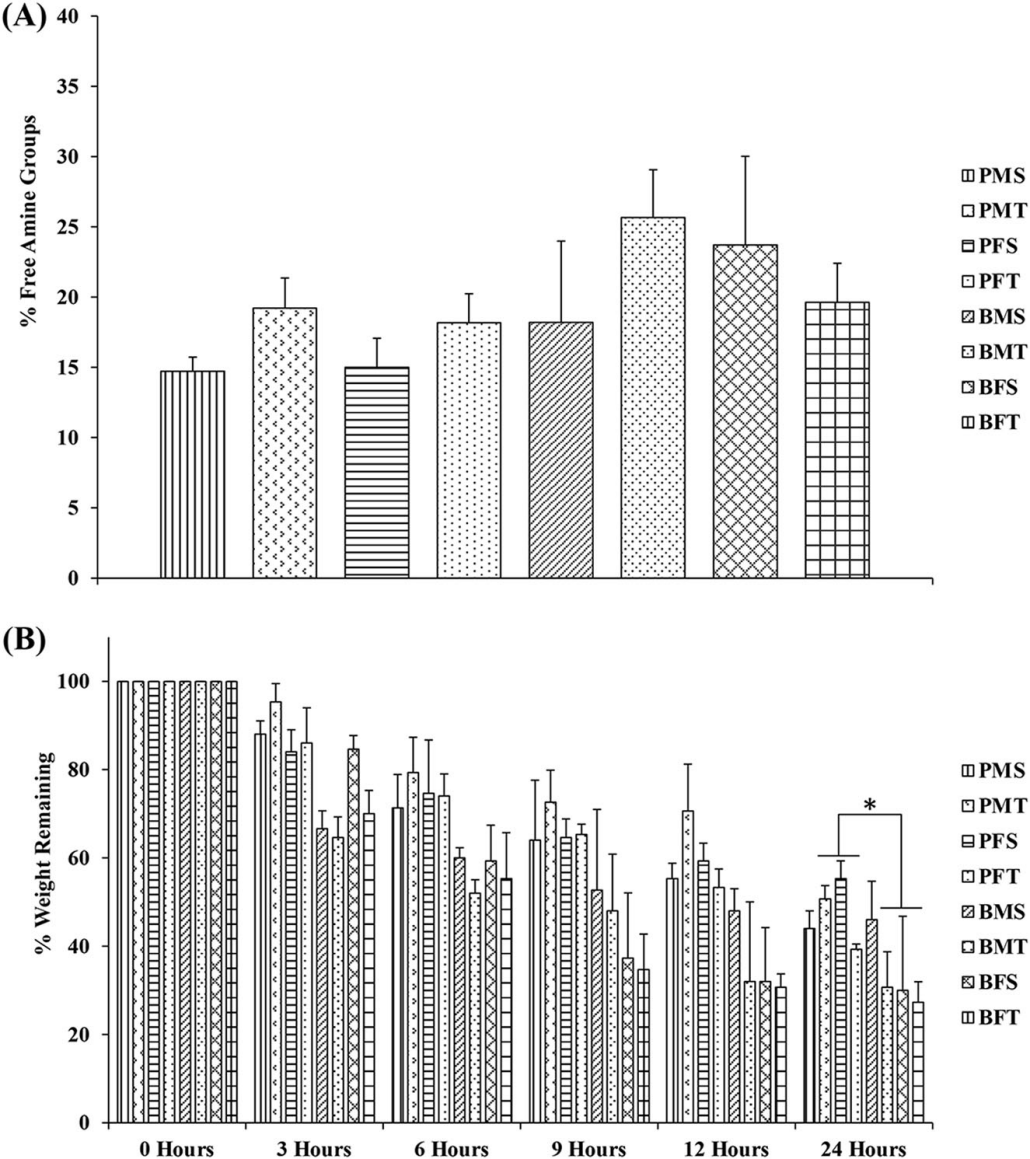
**A** Species, gender and tissue did not significantly (*p* > 0.05) affect free-amine content. *N* = 3. **B** Bovine MT, FS and FT exhibited significantly lower (*p* < 0.01) resistance to collagenase degradation than their porcine counterparts after 24 h of enzyme incubation. *N* = 3. Symbol “*” indicates statistically significant difference

Ninhydrin assay (Fig. 3A) revealed no significant differences (*p* > 0.05) in free-amine content as a function of species, gender and tissue. After 24 h, bovine MT, FS and FT exhibited significantly lower (*p* < 0.01) resistance to enzymatic degradation than their porcine counterparts (Fig. 3B).

### Thermal stability, swelling and mechanical properties analyses

DSC analysis (Table 1) revealed that sponges produced from porcine collagen exhibited significantly higher (*p* < 0.001) denaturation temperature than the bovine collagen sponges of the same gender and tissue, whilst sponges produced from bovine collagen had significantly higher (*p* < 0.001) PBS absorption capacity (Table 1) than porcine collagen sponges of the same gender and tissue. Compression test (Table 1) revealed that sponges produced from porcine collagen exhibited significantly lower (*p* < 0.001) compressive stress and modulus than bovine collagen sponges of the same gender and tissue. Between both species and genders, tendon derived scaffolds exhibited significantly higher (*p* < 0.001) compressive stress and modulus than the skin-derived scaffolds.

**Table 1:** Sponges produced from porcine collagen exhibited significantly higher (*p* < 0.001) denaturation temperature and significantly lower (*p* < 0.001) swelling ratio, compressive stress and modulus than bovine collagen sponges of the same gender and tissue

Group	Peak temperature ± SD (°C)	Swelling ± SD (%)	Compressive stress at 70% deformation ± SD (KPa)	Compressive modulus (Young’s modulus) ± SD (KPa)
PMS	55.43 ± 1.24	1603 ± 439	0.75 ± 0.24	0.63 ± 0.29
PMT	54.99 ± 1.08	498 ± 171	1.09 ± 0.13	1.31 ± 0.19
PFS	53.70 ± 1.36	520 ± 109	0.93 ± 0.62	1.07 ± 0.85
PFT	53.08 ± 1.28	771 ± 226	1.56 ± 0.18	2.29 ± 0.30
BMS	51.03 ± 0.46	1977 ± 463	1.27 ± 0.19	1.78 ± 0.20
BMT	47.65 ± 1.22	7546 ± 1736	2.27 ± 0.49	2.84 ± 0.59
BFS	50.55 ± 1.14	2502 ± 529	1.03 ± 0.21	1.29 ± 0.27
BFT	49.63 ± 1.06	12,031 ± 1900	3.18 ± 0.66	4.67 ± 1.61

Tendon derived scaffolds exhibited significantly higher (*p* < 0.001) compressive stress and modulus than skin-derived scaffolds, independently of the species and gender

Denaturation temperature: *N* = 5; Swelling: *N* = 6; Mechanical properties: *N* = 10

### Dermal fibroblast biological analysis

**Fig. 4:**
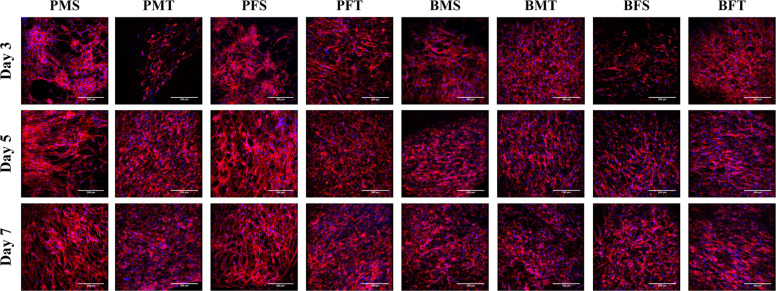
Cellular staining (red: cytoskeleton and blue: nuclei) of human dermal fibroblasts at day 3, 5 and 7 demonstrated that all sponges supported cellular growth independently of species, gender and tissue. *N* = 3

**Fig. 5:**
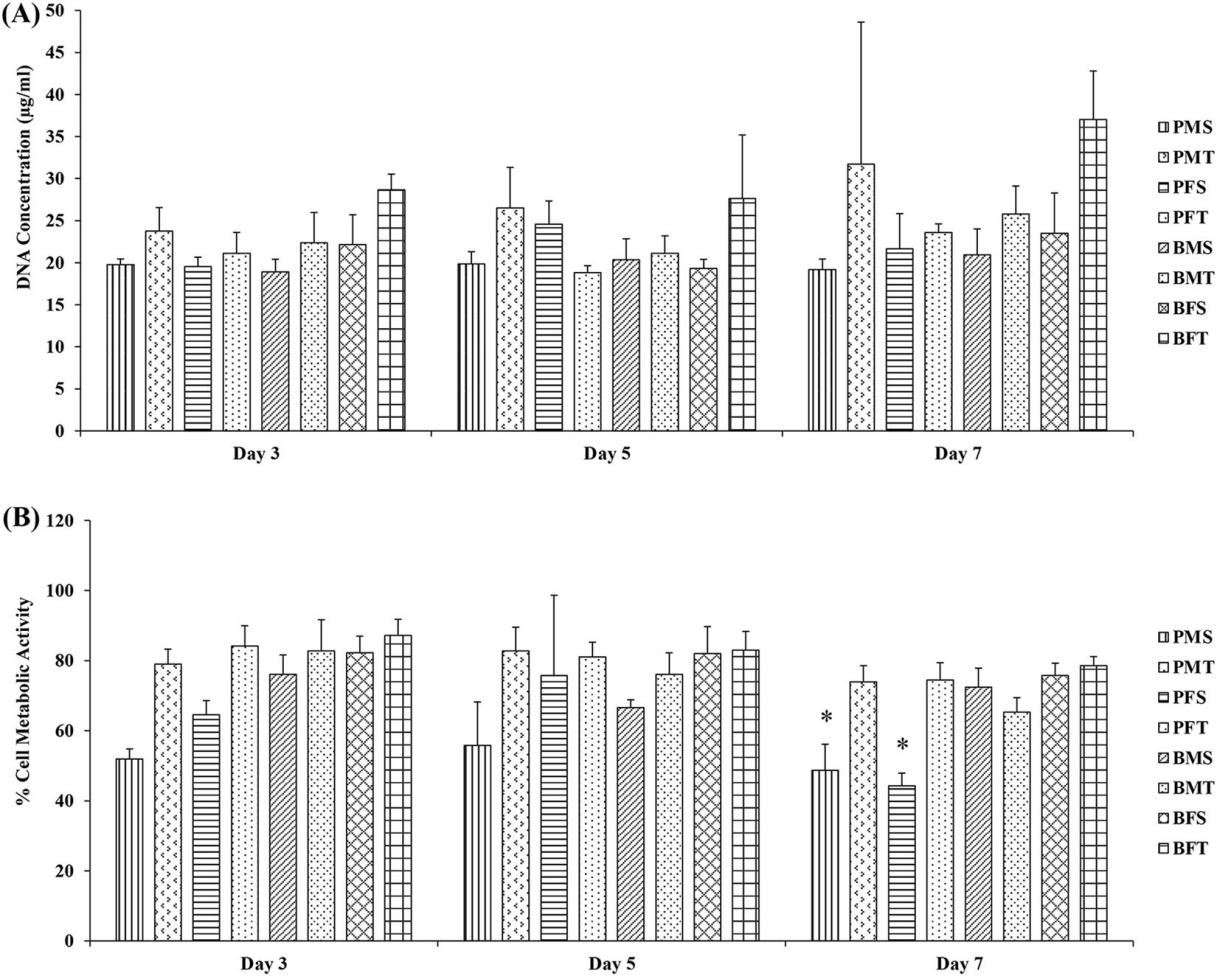
**A** DNA concentration of human dermal fibroblasts at day 3, 5 and 7 was not significantly (*p* > 0.05) affected as a function of species, gender and tissue. **B** By day 7, human dermal fibroblasts grown of PMS and PFS exhibited the lowest (*p* < 0.001) metabolic activity, whilst no significant differences (*p* > 0.05) were observed between the other groups. *N* = 3. Symbol “*” indicates statistically significant difference from all other groups

Cytoskeleton and nuclei staining (Fig. 4) demonstrated that both porcine and bovine collagen sponges supported cellular growth, independently of the tissue and gender. Quantitative morphometric analysis (Supplementary Fig. S1) revealed no apparent differences (*p* > 0.05) in nuclei area and elongation, as a function of species, gender and tissue. Cell viability (Supplementary Fig. S2) and DNA concentration (Fig. 5A) were not affected as a function of species, gender and tissue, whilst PMS and PFS exhibited the lowest (*p* < 0.001) metabolic (Fig. 5B) activity at day 7; there were no significant differences (*p* > 0.05) in metabolic activity (Fig. 5B) between the other groups.

### Monocyte biological analysis

**Fig. 6:**
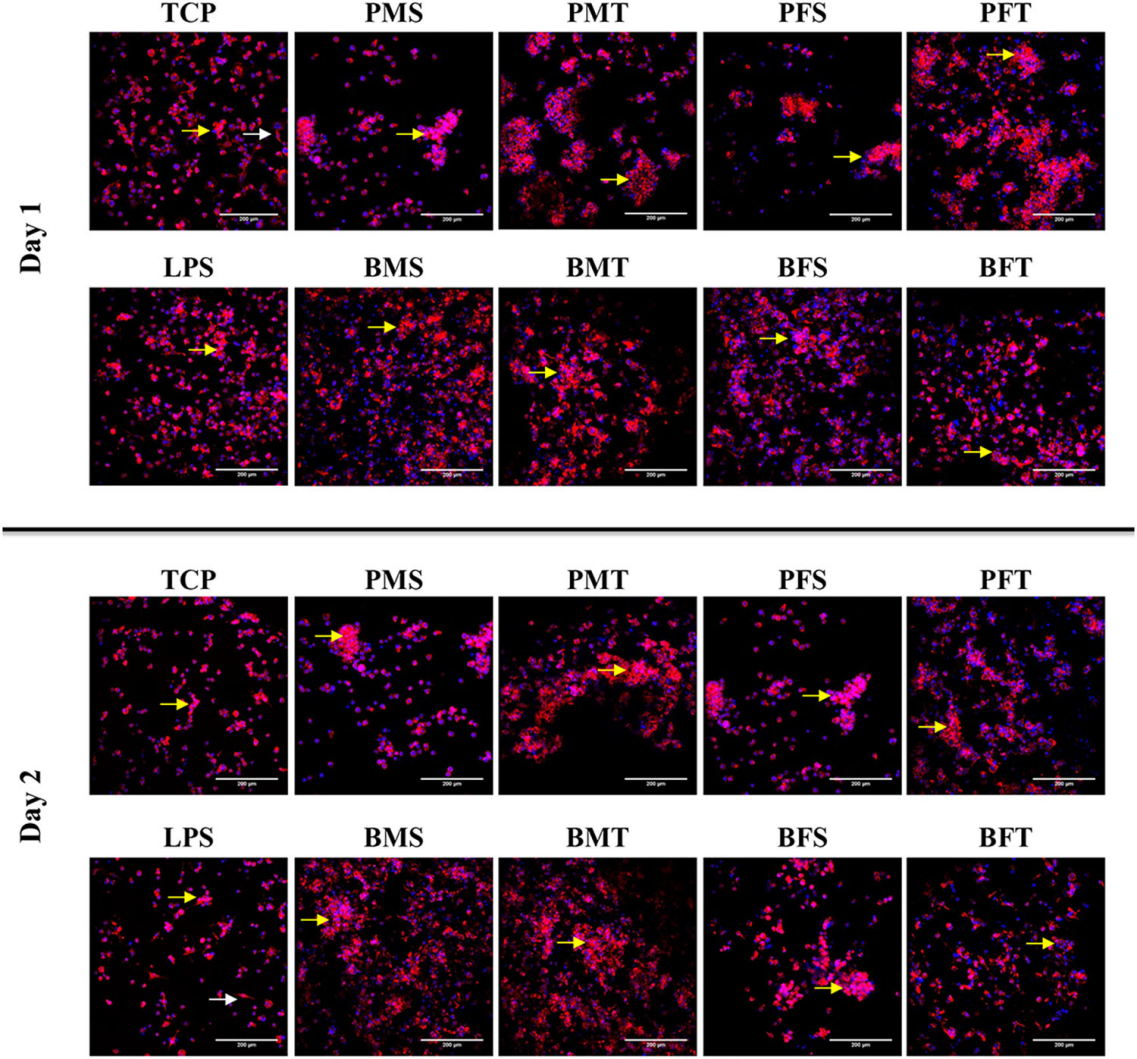
Cellular staining (red: cytoskeleton and blue: nuclei) of THP-1 cells at day 1 and 2 revealed that most cells adopted a rounded morphology and formed aggregates on all samples (five or more cells; yellow arrows), including TCP and LPS controls. Some cells on TCP and LPS also exhibited an elongated morphology (white arrows). *N* = 3

Qualitative cell morphology analysis via cytoskeleton and nuclei staining (Fig. 6) revealed that most of the THP-1 cells adopted a rounded morphology and formed cell aggregates (five or more cells) independently of time point, species, gender and tissue. Although some cells grown on TCP and LPS adopted an elongated cell morphology, most cells exhibited a rounded morphology and also formed aggregates (five or more cells) at both time points.

**Fig. 7:**
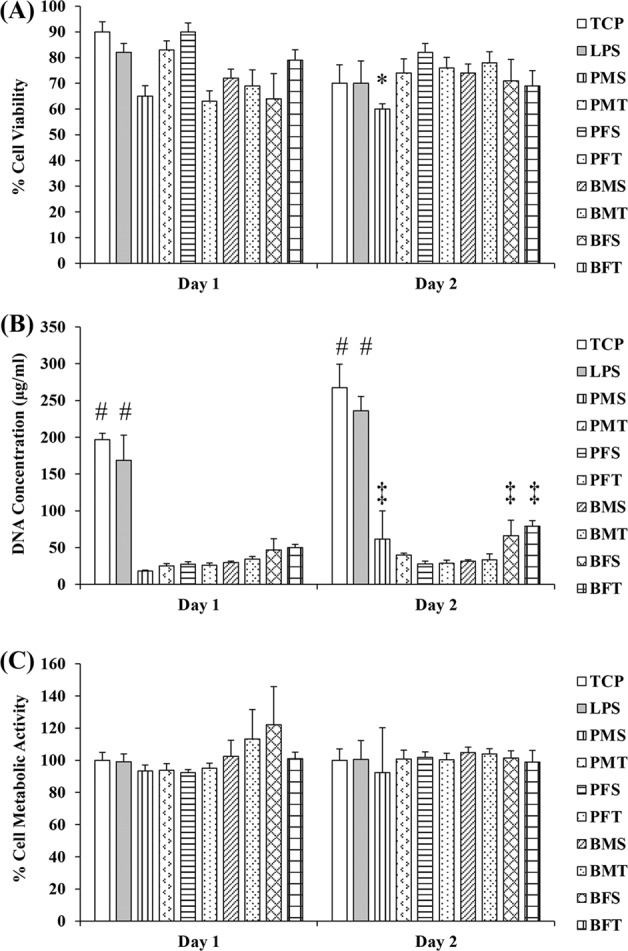
**A** At day 2, among the collagen groups, the PMS sponges exhibited the lowest (*p* < 0.001) THP-1 viability. **B** Add both times point, TCP and LPS has the highest DNA concentration and at day 2, among the collagen groups, the BFT sponges had the highest (*p* < 0.001) THP-1 DNA concentration. **C** No significant differences (*p* > 0.05) were observed between all groups in THP-1 metabolic activity. *N* = 3. Symbol “*” indicates statistically significant difference from all groups; Symbol “#” indicates statistically significant difference from all groups; Symbol “‡” indicates statistically significant difference from collagen groups

At day 2, the PMS exhibited the lowest cell viability (*p* < 0.001), whilst no significant differences were observed between the other groups (*p* > 0.05) (Fig. 7A and Supplementary Fig. S3). Cells on TCP and cells treated with LPS had the highest DNA concentration (*p* < 0.001) and, at day 2, the PMS, BFS and BFT sponges had significantly higher DNA concentration than the other collagen groups (*p* < 0.001) (Fig. 7B). No significant differences were observed in metabolic activity between all groups (*p* > 0.05) (Fig. 7C).

## Discussion

Collagen is the most abundant protein family in vertebrates. Among the 29 collagen subtypes, collagens type I, type II and type III are more frequently encountered and together comprising around 80–90% of the total body collagen [4]. This abundance, along with various properties (e.g. tissue-specific structure and mechanical properties, physiological biodegradability, low antigenicity, cell recognition signals) make collagens the materials of choice for various biomedical applications, including skin [31], bone [32], tendon [33] and cartilage [34] repair and regeneration. Although collagen type I can be extracted from various animals and tissues, type I collagens extracted from skin or tendon of close herd porcine or bovine animals represent the lion’s share. Considering though that collagen is a biological material, differences in amino acid composition as a function of species, age, gender and tissue may affect the properties of the resultant scaffolds. Although collagen extracted from different species and tissues has been shown to produce scaffolds with different properties [7, 12, 35], no study has compared the properties of collagen-based scaffolds as a function of species, gender and tissue. Thus, herein, we assessed the properties of extracted collagen and subsequently produced collagen sponges, as a function of species (porcine and bovine), tissue (skin and tendon) and gender (male and female).

All collagen preparations were produced following a pepsin digestion and a filtration, double salt precipitation, centrifugation and dialysis protocol, which has been shown to increase yield and purity, respectively [12]. In fact, the produced collagen solutions were as pure as the commercially available standard that was used in this study. With respect to yield, the porcine tendons yielded more collagen than the bovine tendons, which can be attributed to the lower level of activity of the porcine tendons (pigs are slaughtered at 100–120 Kg weight, whilst calves are slaughtered at 460–640 Kg) and thus lower cross-linking density and higher solubility [36, 37]. With respect to skin yield, one would have expected more collagen to be extracted from bovine skin as opposed to porcine skin. To substantiate this, we should consider that domesticated pigs evolved from the wild boar and adapted a thick and tightly interwoven collagen network. Indeed, the collagen fibres within the skin of the pig are arranged in two directions, creating an interwoven dense network of small fibres and fibre bundles. The fibres and fibre bundles cross each other and merge from one bundle to the next, with smaller fibres interweaving in between and in different orientations. This compact, higher-order network is also interwoven with elastic fibres [38, 39]. Bovine skin, on the other hand, is thinner in comparison to the porcine skin. The collagen fibres in the bovine skin align in a non-uniform order and it is thought that fibres align parallel to the cows’ spinal column to allow for the skin movement while grazing and walking [40]. However, in our case, the porcine skin groups yielded more collagen than the bovine skin groups, possibly attributed to the age of the animals (pigs are slaughtered at month 5.0–5.5, whilst calves are slaughtered between month 14 and 24). As the animal ages, more mature cross-links are formed that renders collagen solubility [41–44].

Various factors (e.g. freeze-drying parameters, concertation of solution, cross-linking time/density) have been shown to affect pore size and porosity of freeze-dried materials [45–48]. Considering that all freeze-drying parameters and the collagen concentration were kept constant for all collagen preparations, the observed differences may be attributed to the random nature of the process that results in materials with heterogeneous pore structure and with large variations in average pore size throughout the material [49], as opposed to the inherent properties of the collagen preparations. In any case, the produced collagen sponges were highly porous, which is beneficial for cell adhesion, proliferation, migration and differentiation [50–52].

Although no significant differences were observed in free-amine content, as a function of species, gender and tissue, in general, sponges produced from porcine collagen exhibited significantly higher resistance to enzymatic degradation and denaturation temperature and significantly lower swelling ratio than bovine collagen sponges of the same gender and tissue. All these suggest a higher in cross-linking density material, which is surprisingly, considering that the porcine tissues yielded the highest amount of collagen, which indicates lower cross-linking density. As all these properties were assessed with freeze-dried sponges, we believe that these differences may be due to freeze-drying-induced intermolecular cross-linking that provided better organisation and stabilisation of the helices through maintenance of the distances between neighbouring molecules and prevention of incorporation of excess water that disrupts hydrogen and electrostatic bond formation between molecules [53–57]. This has been more profound with the porcine collagen preparations possibly due to the lower extend of cross-linking, as the animals were of younger age.

Mechanical properties wise, porcine collagen sponges exhibited significantly lower compression stress and modulus than bovine collagen sponges of the same gender and tissue and tendon derived scaffolds exhibited significantly higher compression stress and modulus than skin-derived scaffolds, independently of the species and gender. Both these observations may be attributed to the ‘memory’ of collagen from the tissue that has been extracted. Bovine (older animals) extracted collagen sponges had higher mechanical properties than porcine (younger animals) extracted collagen sponges and tendon extracted collagen sponges had higher mechanical properties than skin extracted collagen sponges, as previous studies have shown age-related increases in mechanical properties as a function of increased cross-linking density [58] and increased mechanical properties as a function of weight-bearing tasks [59], respectively. The higher in mechanical properties collagen sponges derived from tendon tissues in comparison to collagen sponges derived from skin tissues can also be attributed to the architecture of the collagen fibres in the respective tissues, which, in turn, is responsible for the tissue-specific biomechanical properties. In skin, collagen fibres are more loosely packed and are interwoven with elastin fibres, whilst in tendons collagen fibres are more closely packed along the longitudinal axis of the tissue, as has been revealed by polarised, transmission electron and second harmonic generation microscopy [60–63].

With respect to biological analysis, in general, all collagen sponges supported human dermal fibroblast attachment, proliferation and growth. The only deviation was observed in (reduced) metabolic activity of human dermal fibroblasts grown on PMS and PFS sponges. It is worth noting though that these sponges had the lowest modulus values and it has been well described in the literature the influence of substrate rigidity on dermal fibroblast growth and function [64–66]. THP-1 morphology analysis revealed that most cells adopted a rounded morphology and formed aggregates, whilst some cells on TCP and LPS groups exhibited an elongated morphology. Rounded cell morphology is associated with M1 pro-inflammatory response, elongated morphology is indicative of M2 anti-inflammatory response phenotype and cell aggregates suggest foreign body response [67–69]. The BFT sponges formed the least aggregates and had the highest DNA concentration; this may be due to the fact that these scaffolds had also the highest modulus values, which may be explained considering that previous studies have associated macrophage response to substrate rigidity [70, 71]. Whether though such slight increase in rigidity is capable of inducing macrophage response has yet to be verified and should be investigated further.

With respect to the influence of gender on the properties of the produced scaffolds, some differences were observed. For example, collagen sponges from porcine female tendon and skin had significantly higher mechanical properties than collagen sponges from porcine male tendon and skin. However, collagen sponges from bovine female tendon had significantly higher mechanical properties than collagen sponges from bovine male tendon and the reverse was the case for skin-derived collagen. Although gender-dependant differences have been documented in the literature for mechanical, structural and compositional properties of tendon [16–21] and skin [22–26], a more detailed investigation (e.g. analysing the properties of the original tissue and the derived scaffolds) is required to safely conclude on the influence of the gender on the properties of the scaffold.

## Conclusions

Collagen type I is the most abundant extracellular matrix protein in vertebrates. This abundance makes collagen the material of choice for scaffold fabrication. Herein we illustrated that although purity, free-amine content and biological (human dermal fibroblast and THP-1 monocyte cultures) response were not affected as a function of species (porcine versus bovine), gender (female versus male) and tissue (skin versus tendon) from which the collagen was extracted, yield, denaturation temperature, resistance to enzymatic degradation, swelling ratio and biomechanical properties were certainly species and tissue dependent. To safely conclude on the influence of gender, more detailed studies are required. Collectively, these data suggest that all these parameters should be considered in the development of a collagen-based implantable device.

## Supplementary Information

Supplementary Information
