# Supplementary material for: The influence of animal species, gender and tissue on the structural, biophysical, biochemical and biological properties of collagen sponges
Source: J Mater Sci Mater Med. 2021 Jan 21;32(1):12. doi: 10.1007/s10856-020-06485-4 (PMC7819930; doi:10.1007/s10856-020-06485-4)

**Supplementary Information**

**Title**

The influence of animal species, gender and tissue on the structural, biophysical, biochemical and biological properties of collagen sponges

**Authors**

Anna Sorushanova ^1, 2^, Ioannis Skoufos ^3^, Athina Tzora ^3^, Anne Maria Mullen ^4^, Dimitrios I. Zeugolis* ^1, 2, 5^

**Affiliations**

1. Regenerative, Modular & Developmental Engineering Laboratory (REMODEL), Biomedical Sciences Building, National University of Ireland Galway (NUI Galway), Galway, Ireland

2. Science Foundation Ireland (SFI) Centre for Research in Medical Devices (CÚRAM), Biomedical Sciences Building, National University of Ireland Galway (NUI Galway), Galway, Ireland

3. Laboratory of Animal Science, Nutrition and Biotechnology, School of Agriculture, University of Ioannina, Arta, Greece

4. Teagasc, Food Research Centre, Ashtown, Dublin, Ireland

5. Regenerative, Modular & Developmental Engineering Laboratory (REMODEL), Faculty of Biomedical Sciences, Università della Svizzera Italiana (USI), Lugano, Switzerland

* Corresponding Author: Dimitrios I. Zeugolis, REMODEL, NUI Galway & USI. Telephone: +41 58 666 40 00; Email: dimitrios.zeugolis@usi.ch

**Supplementary Figure S1:** Quantitative morphometric analysis of human dermal fibroblasts at day 3, 5 and 7 revealed no apparent significant (*p* > 0.05) differences in (**A**) nuclei area and (**B**) nuclei elongation as a function of species, gender and tissue. N=3


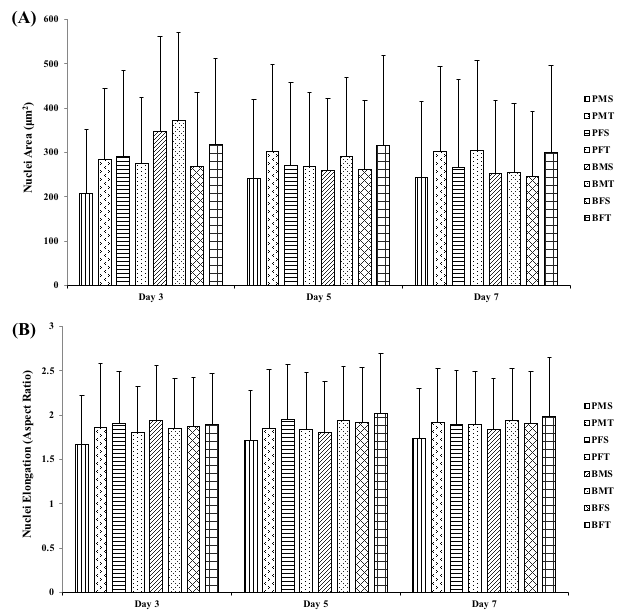


**Supplementary Figure S2:** Cellular viability of human dermal fibroblasts at day 3, 5 and 7 was affected as a function of species, gender and tissue. Green: live cells, Red: dead cells. N=3.


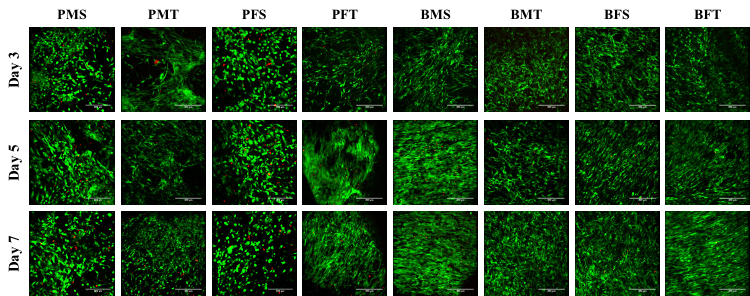


**Supplementary Figure S3:** At day 2, among the collagen groups, the PMS sponges exhibited the lowest (*p* < 0.001) THP-1 viability (quantification is provided at **Figure 7A**). Green: live cells, Red: dead cells. N=3.


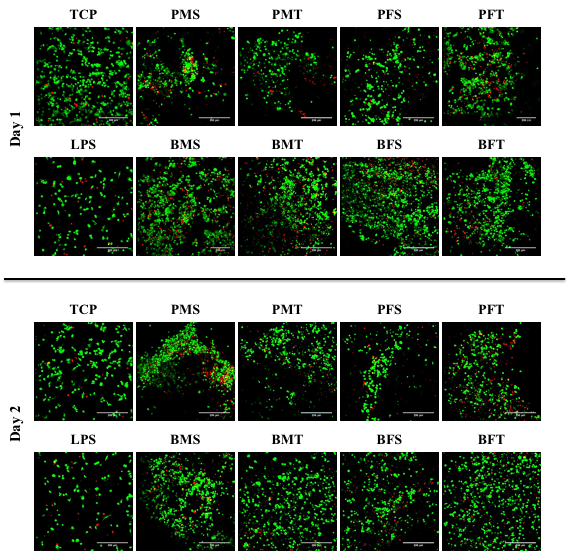

Supplement: Supplementary file 1 — Supplementary Information [file 10856_2020_6485_MOESM1_ESM.docx]
